# Game On: Assessing the Therapeutic Benefits of Board Games in Schizophrenia Management

**DOI:** 10.1192/j.eurpsy.2025.2177

**Published:** 2025-08-26

**Authors:** R. M. Lopes, A. F. Silva, A. C. Rodrigues, A. C. Matias-Martins, F. M. A. Santos, P. M. F. Coelho, T. A. A. Vieira, V. S. Melo

**Affiliations:** 1 Psychiatry and Mental Health Department, Unidade Local de Saúde do Médio Tejo, Tomar, Portugal

## Abstract

**Introduction:**

Schizophrenia is a complex psychotic disorder characterized by positive symptoms (such as delusions, hallucinations, and disorganized speech and behavior), negative symptoms, and cognitive impairment. Cognitive deficits, including impairments in executive function, memory, and social cognition, are particularly persistent and significantly impact daily functioning and overall quality of life. While cognitive remediation has proven effective, recent research has explored the potential of board games as a therapeutic tool.

**Objectives:**

This study aims to assess the therapeutic benefits of board games on cognitive function, specifically executive function, in patients with schizophrenia.

**Methods:**

A literature review was conducted using articles from PubMed, focusing on the terms “board game”, “schizophrenia” and “cognition”.

**Results:**

Cognitive deficits in schizophrenia contribute significantly to poor functional outcomes and daily functioning. Improving cognitive function and social behaviors has been a major focus of psychiatric rehabilitation techniques. Board games have been found to improve various aspects of cognitive function, including attention, working memory, speed of processing, verbal learning, visual learning, reasoning and problem solving, as well as social cognition. They are expected to enhance knowledge, interpersonal interactions, and increase participant motivation. Ideally, an effective board game for this population should be behaviorally oriented, emphasize positive reinforcement and shaping, and be sensitive to cognitive limitations through repetition and procedural learning. Additionally, they should be engaging and fun to address negative symptoms.

**Conclusions:**

Board games present a promising therapeutic avenue for managing schizophrenia, particularly in enhancing cognitive function and social skills. While cognitive remediation programs have already demonstrated efficacy, board games offer a more accessible and engaging alternative. Despite these positive findings, the limited number of studies and inconsistent long-term data highlight the need for further research. Future studies should evaluate the durability of cognitive and functional improvements from board game interventions and explore their integration into comprehensive treatment programs for schizophrenia.

**Disclosure of Interest:**

None Declared

